# Toward Preparing a Knowledge Base to Explore Potential Drugs and Biomedical Entities Related to COVID-19: Automated Computational Approach

**DOI:** 10.2196/21648

**Published:** 2020-11-10

**Authors:** Junaed Younus Khan, Md Tawkat Islam Khondaker, Iram Tazim Hoque, Hamada R H Al-Absi, Mohammad Saifur Rahman, Reto Guler, Tanvir Alam, M Sohel Rahman

**Affiliations:** 1 Department of Computer Science and Engineering Bangladesh University of Engineering and Technology Dhaka Bangladesh; 2 College of Science and Engineering Hamad Bin Khalifa University Doha Qatar; 3 International Centre for Genetic Engineering and Biotechnology, Cape Town Component Cape Town South Africa; 4 Division of Immunology and South African Medical Research Council Immunology of Infectious Diseases, Department of Pathology, Institute of Infectious Diseases and Molecular Medicine Faculty of Health Sciences University of Cape Town Cape Town South Africa; 5 Wellcome Centre for Infectious Diseases Research in Africa, Institute of Infectious Disease and Molecular Medicine Faculty of Health Sciences University of Cape Town Cape Town South Africa

**Keywords:** COVID-19, 2019-nCoV, coronavirus, SARS-CoV-2, SARS, remdesivir, statin, statins, dexamethasone, ivermectin, hydroxychloroquine

## Abstract

**Background:**

Novel coronavirus disease 2019 (COVID-19) is taking a huge toll on public health. Along with the non-therapeutic preventive measurements, scientific efforts are currently focused, mainly, on the development of vaccines and pharmacological treatment with existing drugs. Summarizing evidences from scientific literatures on the discovery of treatment plan of COVID-19 under a platform would help the scientific community to explore the opportunities in a systematic fashion.

**Objective:**

The aim of this study is to explore the potential drugs and biomedical entities related to coronavirus related diseases, including COVID-19, that are mentioned on scientific literature through an automated computational approach.

**Methods:**

We mined the information from publicly available scientific literature and related public resources. Six topic-specific dictionaries, including human genes, human miRNAs, diseases, Protein Databank, drugs, and drug side effects, were integrated to mine all scientific evidence related to COVID-19. We employed an automated literature mining and labeling system through a novel approach to measure the effectiveness of drugs against diseases based on natural language processing, sentiment analysis, and deep learning. We also applied the concept of cosine similarity to confidently infer the associations between diseases and genes.

**Results:**

Based on the literature mining, we identified 1805 diseases, 2454 drugs, 1910 genes that are related to coronavirus related diseases including COVID-19. Integrating the extracted information, we developed the first knowledgebase platform dedicated to COVID-19, which highlights potential list of drugs and related biomedical entities. For COVID-19, we highlighted multiple case studies on existing drugs along with a confidence score for their applicability in the treatment plan. Based on our computational method, we found Remdesivir, Statins, Dexamethasone, and Ivermectin could be considered as potential effective drugs to improve clinical status and lower mortality in patients hospitalized with COVID-19. We also found that Hydroxychloroquine could not be considered as an effective drug for COVID-19. The resulting knowledgebase is made available as an open source tool, named COVID-19Base.

**Conclusions:**

Proper investigation of the mined biomedical entities along with the identified interactions among those would help the research community to discover possible ways for the therapeutic treatment of COVID-19.

## Introduction

SARS-CoV-2 initially spread widely in China, then in Italy, and has since been reported worldwide [1,2]. SARS-CoV-2 is a novel coronavirus that causes COVID-19 [3]. Although SARS-CoV-2 has gained attention as a consequence of the global COVID-19 pandemic, other known human coronaviruses, including betacoronaviruses (SARS-CoV, MERS, OC43, HKU1) and alphacoronaviruses (229E, NL63), have resulted in severe respiratory syndrome in patients and been of public health concern [4]. To combat COVID-19, an urgent solution is needed for the detection and therapeutic treatment of this disease, which requires a comprehensive experimental investigation of relevant biomedical entities (eg, genes, noncoding ribonucleic acids [ncRNA], viruses, drugs) [5]. However, this is a relatively slow process due to the inherent nature of experimental validation. As an alternative, faster in silico methods can be applied [6,7], which can act as a filter prior to wet lab validation. Virtual screening, molecular docking, and other in silico methods have already been investigated to discover drugs that may work against COVID-19 [8]. Still, this is a daunting task due to the large number of possible combinations of biomedical entities (eg, drug-gene pairs) that need to be examined [9]. To enable comprehensive exploration of potential therapeutic treatments, knowledge base solutions are proposed; these would allow the scientific community to focus on a relatively smaller number of potential biomedical entities that may lead to the discovery of a novel treatment for COVID-19.

Databases that focus on virus-related diseases for multiple hosts already exist. For example, in ViRBase [10], the authors highlighted the association between ncRNAs and viruses in 20 hosts. The VISDB database, based on literature mining, integrated the virus interaction site in humans for five DNA oncoviruses and four RNA retroviruses [11]. Virus Pathogen Resources (VIPR) developed a portal that collected a comprehensive set of information related to coronavirus and hepatitis C virus (HCV), as well as other viruses [12,13]. However, none of the abovementioned databases are particularly useful for COVID-19/SARS-CoV-2, as those databases were not specific to the novel coronavirus, or they provided very limited information about the associated genes, or they did not include other factors involved in coronavirus-related diseases, drugs, and drug side effects. Moreover, there is no one knowledge base that has integrated all biomedical entities specific to COVID-19/SARS-CoV-2. To address this gap, we explored the potential of machine intelligence to automatically mine the scientific literature, with the goal of developing the first comprehensive knowledge base that integrates several biomedical entities associated with COVID-19/SARS-CoV-2. To achieve this, we leveraged state-of-the-art natural language processing algorithms, sentiment analysis, and deep learning–based techniques and applied them to a large corpus of coronavirus-related scientific literature.

## Methods

### Data Sets

For this study, we used the COVID-19 Open Research Dataset (CORD-19) [14], generated by the Allen Institute for AI. The data set contains over 138,000 scholarly articles related to COVID-19 and the coronavirus family of viruses. The data set was collected using the following query to search PubMed, PubMed Central (PMC), bioRxiv, and medRxiv: “COVID-19” OR “Coronavirus” OR “Corona virus” OR “2019-nCoV” OR “SARS-CoV” OR “MERS-CoV” OR “Severe Acute Respiratory Syndrome” OR “Middle East Respiratory Syndrome.” This query covers most research articles related to COVID-19 and other coronaviruses (eg, MERS, SARS) and we searched up until June 9, 2020. Unless otherwise specified, we considered both the abstract and full body of the manuscripts (when available) for downstream analysis.

### Source of Dictionaries

We collected gene names from HUGO Gene Nomenclature Committee (HGNC) [15], Protein Data Bank (PDB) entries from PDB [16], micro ribonucleic acids (miRNAs) from miRBase [17], disease names from Disease Ontology (DO) [18], drug names from DrugBank [19], and drug side effects from Side Effect Resource (SIDER) [20].

### Overview of Methodology

We extracted disease-drug, disease-gene, drug-PDB pairs, and their corresponding sentences from the CORD-19 literature in a co-occurrence–based approach. To evaluate the effectiveness of the disease-drug pairs, we used both a pretrained model (TextBlob) and an unsupervised model (developed by the authors using the Word2Vec model and K-means clustering) to determine the sentiment scores of the sentences extracted for each pair. We further used these sentiment scores along with the minimum distance between the disease and drug term in the corresponding sentences as input features of our neural network model, which we used for the final classification of the disease-drug pairs (as positive or negative). To determine the confidence level of the extracted disease-gene associations, we transformed each disease and gene of a pair into two separate vectors using the Word2Vec model and calculated their cosine similarity. We used the known disease-gene associations from the DisGeNET database as the gold standard to determine the confidence level of the new associations on the basis of cosine similarity measures. Finally, we extracted the side effects of the drugs that were found by our mining from SIDER. Additionally, a feedback mechanism was incorporated into COVID-19Base to collect feedback from users for future use.

### Extracting Disease-Drug Interactions

**Figure 1:**
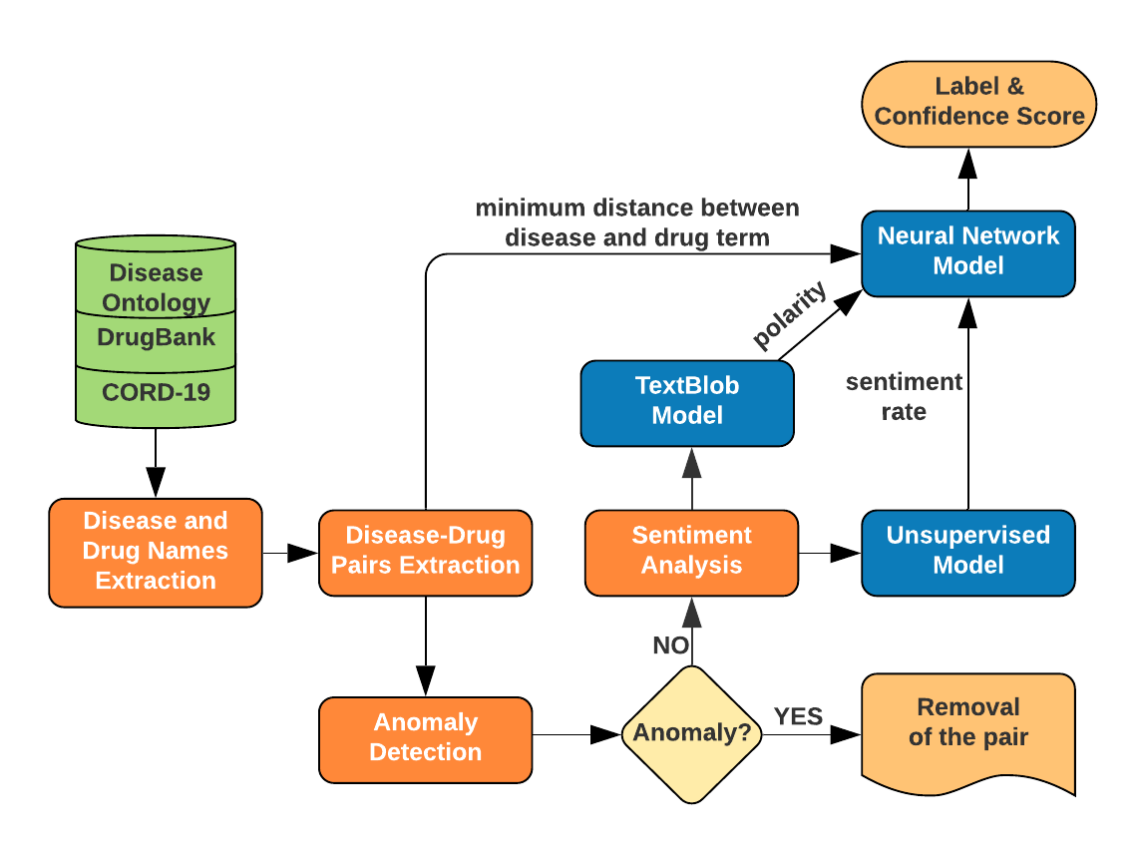
Flowchart of extracting disease-drug interactions and predicting the effectiveness of drugs against diseases with confidence scores. CORD-19: COVID-19 Open Research Dataset.

We extracted disease-drug interactions from the CORD-19 literature and classified them into one of two categories (labels): positive and negative. The positive label means the drug is potentially effective against COVID-19, and the negative label means the opposite. We also determined a confidence score, which indicates our level of confidence in that automatic label. Figure 1 shows the workflow of extracting disease-drug interactions and predicting the effectiveness of drugs against diseases with confidence scores.

### Disease and Drug Name Extraction

To extract relevant disease-drug pairs from the CORD-19 literature, we employed a dictionary-based approach to detect mentions of diseases and drugs in the literature. We used Disease Ontology [18] and DrugBank [19] to prepare the disease and drug dictionaries. We leveraged the Aho-Corasick algorithm [21] to search the drug and disease names, considering the large size of the drug and disease dictionaries and the corpus itself. The Aho-Corasick algorithm is a string-searching algorithm that efficiently locates multiple patterns in a large amount of text. The time complexity of the algorithm is *O(n + m + z)*, where *n* is the length of the text, *m* is the total length of all the patterns to be searched, and *z* is the total number of occurrences of the patterns in the text.

### Disease-Drug Pairs Extraction

After extracting the disease and drug names separately, we wanted to mine the literature and identify the sentences that contain the disease and drug pairs to semantically evaluate their interactions. For this purpose, we searched for every disease-drug pair from our disease and drug list in the CORD-19 literature and collected every sentence where a co-occurrence was found. We then created a document for every disease-drug pair, combining all extracted sentences. Thus, we built a disease-drug pair to document mapping. We did not use a pattern-based approach here (as was done previously in [22]) as this could result in missing some sentences containing disease-drug pairs.

### Anomaly Removal

As we automatically extracted the sentences containing the disease-drug pairs, there was a possibility of errors in our extracted data; therefore, we decided to check and remove any abnormalities from our collected data before moving on to the next stage of the pipeline. We used unsupervised anomaly detection [23] for this task. Unsupervised anomaly detection detects anomalies in an unlabeled data set by looking for instances that seem to fit the remainder of the data set the least, under the assumption that the majority of the instances in the data set are “normal.” We used the K-means clustering algorithm [24], as it has been used for anomaly detection in several studies [25-29]. We proceeded as follows. First, we used Doc2Vec [30] to create a numeric representation of each document associated with each disease-drug pair. We then fitted these representations into our K-means model and observed two clear clusters of easily discriminable sizes, where the smaller one consisted of only 189 instances. As we know that anomalies differ from the normal instances significantly and occur very rarely in the data, we could assume that the instances of the smaller cluster were indeed anomalies. We also checked a number of instances manually to verify our assumption. We discarded these 189 instances from any further consideration.

### Sentiment Analysis

#### Overview

We applied sentiment analysis to automatically assess the effectiveness of a drug to treat a particular disease in the context of each extracted drug-disease pair. First, we applied the concept of transfer learning. We used TextBlob [31], which is a pretrained sentiment analysis tool provided as a Python library. However, it showed some inconsistency in some cases as expected from a pretrained model and we felt it necessary to perform unsupervised sentiment analysis, which is the second model in our pipeline. We obtained a polarity score from the TextBlob model and a sentiment rate from our unsupervised model for each disease-drug pair, which were subsequently fed to our neural network model to predict the final label.

#### TextBlob Model

TextBlob is a Python library that is widely used in natural language processing tasks such as part-of-speech (POS) tagging, noun phrase extraction, sentiment analysis, classification, and translation. Given the sentences that we mined for each disease-drug pair as input, TextBlob gives a polarity score between –1 and 1. We recorded the polarity scores for each disease-drug pair to use it as a feature for our neural network model.

#### Unsupervised Model

We used the concept of K-means clustering again for unsupervised sentiment analysis. First, we trained the Word2Vec [32] model with our mined literature and got a vector representation of every word. We then ran K-means clustering on the estimated word vectors and found two clusters (positive and negative). The positive cluster was decided on the basis of the presence of several positive words (in the context of a disease-drug pair), including “cure,” “preclude,” “inhibit,” “prescribe,” “reduce,” and “modest.” On the other hand, the negative cluster contained words like “risky,” “kill,” and “danger.” We then assigned each word a sentiment value, either +1 or –1, based on the cluster (positive or negative) they belong to. We weighed this value by dividing it by the distance between the word and the centroid of its cluster to describe the extent of its potential positiveness or negativeness. We then calculated the term frequency–inverse document frequency (tf–idf) score [33,34] of each word in the sentence collection to consider the significance of the unique words. Next, we built a tf–idf representation, *T*, for each disease-drug pair by replacing each word of the corresponding sentences with its tf–idf score and a sentiment value representation, *S,* by replacing each word with its sentiment value. Finally, we took their dot product (*T*▪*S*) as the final sentiment rate of our unsupervised model.

### Neural Network Model for Automatic Labeling and Confidence Score

#### Overview

We used a deep neural network (DNN) model to automatically predict the label and confidence score for our disease-drug pairs. We used a relatively simpler neural network with two hidden layers as such models commonly perform better for smaller data sets compared to neural networks with many layers and parameters [35,36].

#### Training Data

We manually labeled 200 disease-drug pairs to train our neural network model. Among them, there were 110 positive instances and the rest were negative.

#### Input Features

We used the polarity or sentiment score given by the TextBlob and unsupervised models as the input features for our neural network model, along with the minimum distance between the disease and drug term in the corresponding document.

#### Model Setup and Output

**Figure 2:**
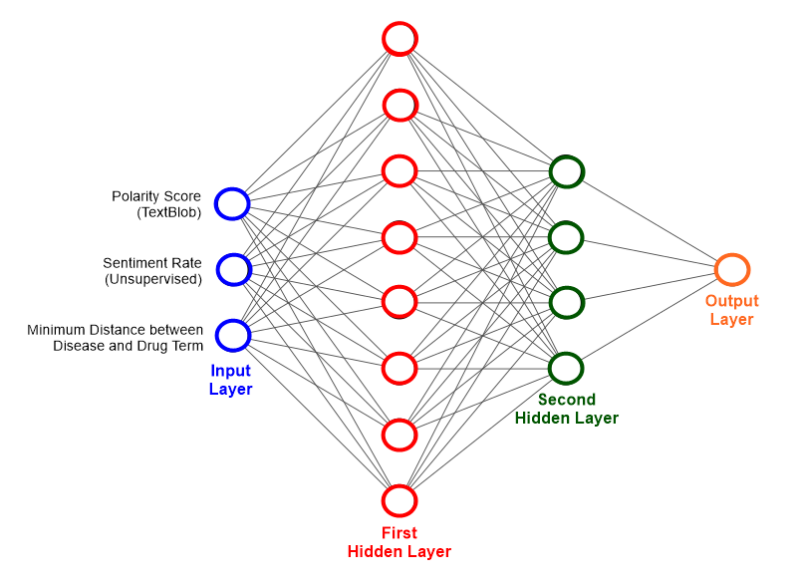
Schematic diagram of the deep neural network used to predict the effectiveness of drugs against diseases.

The DNN structure used in this study is similar to that shown in Figure 2. It consists of one input layer with three neurons (each neuron corresponds to one input feature), two hidden layers with eight and four neurons respectively, and one output layer containing one neuron for binary classification (positive or negative). The transfer functions of the first and second hidden layers were the rectified linear unit (ReLU) [37] and hyperbolic tangent function (tanh) [38], respectively. The transfer function of the output layer was a sigmoid function [39]. We trained the DNN model using Xavier initialization [40], which tries to make the variance of the outputs of a layer equal to the variance of its inputs. We used Adam optimizer [41] and the maximum training epoch was set to 500. We split our labeled data into training and test sets on an 80:20 ratio. We trained our model on the training data and achieved 75% accuracy on the test set.

### Extracting Disease-Gene Associations

**Figure 3:**
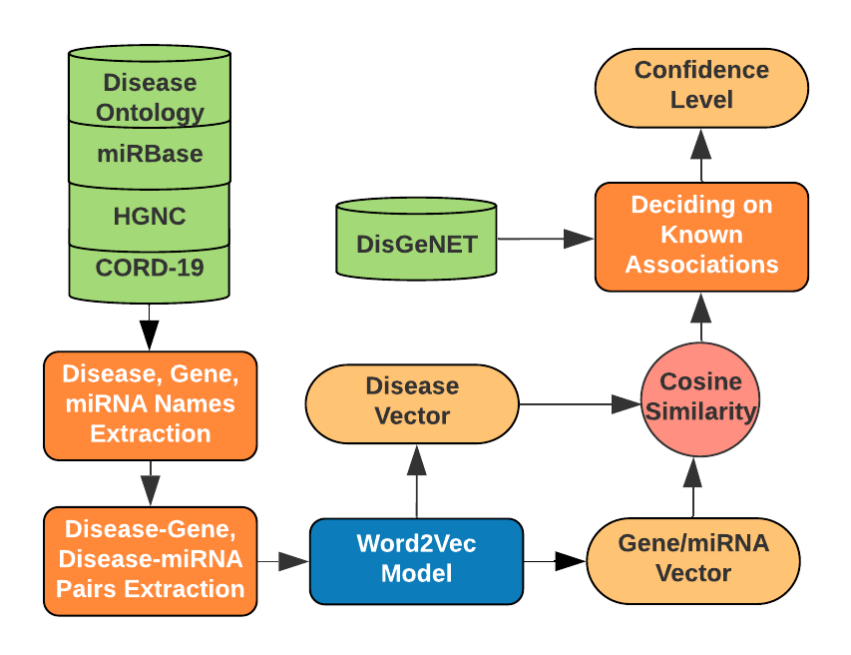
Flowchart of extracting disease-gene and disease-miRNA associations and determining their confidence levels. CORD-19: COVID-19 Open Research Dataset; HGNC: HUGO Gene Nomenclature Committee; miRNA: micro ribonucleic acid.

Figure 3 shows the workflow of extracting disease-gene associations. We extracted gene names along with miRNAs from the CORD-19 literature in a dictionary-based approach using HGNC [15] and miRBase [17]. We then extracted their associations with diseases in a similar process to the one we had used to extract the disease-drug pairs and collected all the abstracts where a co-occurrence was found. Next, we applied the concept of cosine similarity [42] to confidently infer the associations. We transformed each disease into vector *V_1_*, each gene (and miRNA) into vector *V_2_*, and then calculated the cosine similarity of *V_1_* and *V_2_* for each pair. To create the vector representations, we trained a Word2Vec model with all the collected abstracts. We used the DisGeNET [43] database as the gold standard to evaluate the performance of cosine similarity in predicting the gene-disease linkage. First, we calculated the maximum, average, and minimum cosine similarity of the pairs that were common both in our findings and in the DisGeNET database. We found that 99.7% of the newly discovered pairs lie within this range (determined from DisGeNET) in terms of cosine similarity. We further classified the associations into three classes (high, medium, and low) in terms of confidence as follows: pairs having cosine similarity closest to the maximum (minimum) of the known ones were considered as high (low) confidence associations, and the remaining ones (those closest to the average) as medium confidence associations. Moreover, pairs that were also found in the DisGeNET database were labeled as verified associations.

### Extracting Drug-Protein Associations

We also extracted drug-protein associations from the CORD-19 literature, applying the same co-occurrence–based approach as mentioned above. We used PDB IDs from the Protein Data Bank [16] for extracting protein names. Unlike the disease-gene associations, we did not apply the concept of cosine similarity here as we did not find any suitable data set that could be used as the gold standard in this case.

### Extracting Side Effects of Drugs

The drugs we are suggesting through this literature mining may come with different side effects. Therefore, we also explored the possible side effects of the drugs. We collected the drugs with their corresponding side effects from SIDER [20] and mapped them with the drugs mentioned in the CORD-19 literature to extract the possible side effects.

### Feedback Mechanism

We implemented a feedback mechanism in COVID-19Base for future improvement. This mechanism enables expert users from the scientific community to share their valuable feedback on the label (positive or negative) for a particular interaction determined by the automatic natural language processing–based approach. The users can voluntarily label each sentence that is mined from the literature as a source of an interaction. This feedback will be recorded and further processed to enrich the labeled data set, which can be leveraged in the next version of COVID-19Base to further improve the prediction quality for determining effective disease-drug interactions. The accompanying tutorial (user manual) on COVID-19Base highlighted an example of how a user can use the feedback mechanism.

## Results

### Terms and Interactions Highlighted in CORD-19 Data Set

Based on our computational workflow, we identified 1805 diseases, 2454 drugs, 1910 genes, 11 miRNAs, and 70 PDB entries from the CORD-19 literature (Table 1). Among the disease-drug pairs, 21,581 were positive and 1318 were negative. Among the disease-gene associations, 2088 were verified, and 82 associations were found with high-confidence, 12,231 with medium-confidence, and 1488 with low-confidence. More results are shown in Table 1. Notably, a small proportion (1.5%) of the findings were manually labeled. Interestingly, we found 194 drug-PDB pairs for coronavirus-related diseases, which indicates the rapid growth of experimental work to understand the interaction mechanisms of drugs and target proteins.

**Table 1:** Pairs of terms as identified in the analyzed set of documents^a^.

Interaction or association	Number of extracted pairs of terms
Disease-drug	22,899 (21,581 positive, 1318 negative)
Disease-gene	15,889 (2088 verified, 82 high, 12,231 medium, 1488 low)
Disease-miRNA	56 (48 medium, 8 low)
Drug-Protein Data Bank	194

^a^Positive (negative) indicates an (in)effective association. High, medium, and low refer to confidence associations.

### COVID-19–Related Terms and Interactions

Our computational workflow identified 514 drugs and 417 genes that are directly associated with COVID-19 (Table 2). Among the 514 drugs, 492 were found to have a positive association and 22 had a negative association. Among the 417 genes, 347 were medium-confidence associations and 70 were low-confidence associations.

**Table 2:** Biomedical terms that are related to COVID-19^a^.

Interaction or association	Number of extracted pairs of terms
COVID-19–drug	514 (492 positive, 22 negative)
COVID-19–gene	417 (347 medium, 70 low)
COVID-19–miRNA	3 (2 medium, 1 low)

^a^Positive (negative) indicates an (in)effective association. High, medium, and low refer to confidence associations.

### Genes Related to COVID-19

Our automated workflow identified C-reactive protein (CRP) as one of the COVID-19–associated genes with “medium” confidence. CRP is a known clinical biomarker for SARS [44] and the level of CRP increases significantly in patients with SARS. The level of CRP was also higher for patients with COVID-19 in some clinical cases [45,46]. More than 25 papers (from the CORD-19 data set) related to the association between CRP and COVID-19 were identified through our computational workflow. Furthermore, the genes *ELANE*, *AZU1*, *MPO*, *PRTN3*, *CTSG*, and *TCN1* were shown to be significantly altered in patients with COVID-19 [47], and our automatically prepared knowledge base highlights all of them as associated with COVID-19 with “medium” or “low” confidence. The *ACE2* and *TMPRSS2* genes are known to be involved in SARS-CoV-2 infection [48]; in fact, SARS-CoV-2 uses angiotensin-converting enzyme 2 (ACE2) as a receptor for entry into host cells [49,50]. The spike protein of SARS-CoV-2 binds with the ACE2 receptor and the protease TMPRSS2 mediates the infection process [51]. It is important to note that *ACE2* and *TMPRSS2* were not directly listed in DisGeNET as genes associated with COVID-19. In spite of that, our data-driven approach based on a gold-standard data set from DisGeNET was able to infer the association of *ACE2* and *TMPRSS2* with COVID-19 with “medium” confidence, which suggests that our approach is efficacious. Analyzing the complete *ACE2* interaction network, Wicik et al [52] listed several element genes (*ACE2*, *ANPEP*, *DPP4*, *CCL2*, *MEPIA*, *TFRC*, *ADAM17*, *NPC1*, *FABP2*, *TMPRSS2*, *CLEC4M*) and all of these genes were identified as COVID-19–associated in our automatically prepared knowledge base. In addition, we mined three miRNAs (hsa-miR-4661-3p, hsa-miR-429, and hsa-miR-183) that were mentioned in the abstracts of COVID-19–related literature.

### Case Studies

In this section, we discuss interesting and useful findings from our automatically prepared knowledge base in the context of potential drugs that can be investigated for the potential therapeutic treatment of COVID-19.

#### Case Study 1: Dexamethasone Can be Considered an Effective Drug for COVID-19

Dexamethasone, an inexpensive and commonly used steroid, is a major breakthrough in the fight against COVID-19. We found dexamethasone to be a positive (ie, effective) drug for COVID-19, automatically labeled as such through our computational workflow with a confidence score of 77.61%. Our computational workflow also discovered the effectiveness of this drug against pneumonia, respiratory failure, and diarrhea, which are strongly correlated to COVID-19 [53,54]. Thus, further exploration of this drug to fight COVID-19 is likely to be fruitful. Recent studies suggest that dexamethasone reduces the risk of death from COVID-19 from 40% to 28% for patients on ventilators and from 25% to 20% for patients needing oxygen [55].

#### Case Study 2: Ivermectin Might be Considered an Effective Drug for COVID-19

Ivermectin is an effective drug against pneumonia and diarrhea, and has recently been claimed to successfully treat patients with COVID-19 as well [56]. It is a US Food and Drug Administration (FDA)–approved drug used for parasitic infections, which has the potential to be repurposed. Ivermectin inhibits the replication of SARS-CoV-2 in vitro [57]. Recently, a team of medical doctors in Bangladesh reported quick recoveries of patients with COVID-19 using this drug [58]. We found ivermectin to be a positive (ie, effective) drug for COVID-19, automatically labeled with a confidence score of 77.91%. It was also labeled a positive drug for pneumonia and diarrhea in our knowledge base.

#### Case Study 3: Remdesivir Seems Effective Against COVID-19

Remdesivir has been identified as a positive (ie, effective) drug for COVID-19, automatically labeled as such through our pipeline, with a confidence score of 68.18%. Thus, it seems to be a promising drug for further investigation for treating COVID-19. Interestingly, it was recently being considered as an effective drug for treating COVID-19 [59]. Notably, remdesivir is an antiviral drug originally developed for Ebola treatment [60,61]. A recent clinical trial conducted by the National Institute of Allergy and Infectious Diseases (NIAID) showed that remdesivir helped patients with COVID-19 recover faster and improved their survival rates. Adult patients treated with remdesivir were found to recover 4 days faster, an improvement of 31% compared to other patients; in addition, the overall death rate dropped from 11.6% to 8% [62]. Remdesivir is now under consideration for use against COVID-19 in more than ten clinical trials [63]. We found 6LU7 was one of the PDB entries for remdesivir. After exploring the corresponding literature [64], we found that remdesivir was shown to be an effective inhibitor of the main SARS-CoV-2 protease using molecular docking [65,66].

#### Case Study 4: Hydroxychloroquine Is Not an Effective Treatment for COVID-19

Antimalaria drug hydroxychloroquine, which is one of the most talked-about drugs for treating COVID-19, was also found in our mining, albeit with a negative interaction. Our model found it is a negative (ie, ineffective) drug with 64.67% confidence. Additionally, it also revealed that this drug has 111 side effects including anemia, hemorrhage, liver disorder, hepatitis fulminant, cardiomyopathy, and cardiac failure, which makes it a risky option, especially for patients with heart and liver complications. Although the FDA had previously granted authorization to use this drug for COVID-19, it has recently cautioned against its use outside of a hospital setting or a clinical trial due to its side effects and risk factors [67].

#### Case Study 5: Statins Drugs Could be Effective Against COVID-19

Statins are effective as lipid-lowering drugs and mainly used for the treatment of cardiovascular diseases [68]. Statins are also well known for their anti‐inflammatory effects [69] and some studies have supported the use of these drugs as part of a COVID‐19 treatment protocol [70]. Multiple clinical trials (eg, NCT04343001, NCT04380402) have been launched to determine the efficacy of statins against COVID-19 [71,72]. In our knowledge base, the majority of statin classes were shown to be effective against COVID-19. For example, ulinastatin, rosuvastatin, fluvastatin, and lovastatin were labeled as positive (ie, effective) drugs against COVID-19 with 94.04%, 79.38%, 78.88%, and 70.75% confidence scores, respectively. Through our automated computational workflow, we found only one mention of atorvastatin in the literature [73]. In that single article, Deliwala et al [73] mentioned atorvastatin as part of a prevention plan against cortical stroke for a 31-year-old female patient with COVID-19, without referring to the effectiveness of atorvastatin against COVID-19. Consequently, our knowledge base labeled atorvastatin with a negative sentiment and a rather low confidence score (61.22%) for COVID-19. We anticipate that as the number of articles related to atorvastatin use in COVID-19 treatment protocols increases, our model will be able to effectively infer the sentiment (effective versus ineffective) of this drug. Based on our finding, it is safe to state that statins, as low-cost and well-tolerated drugs, should be investigated in more detail in clinical trials; such drugs may help low- and middle-income countries in particular, where expensive drugs might not be affordable.

## Discussion

### Principal Findings

In our knowledge base, through a computational workflow, we not only extracted the drugs and other biomedical terms that are mentioned in the literature, but also identified “term pairs” based on their co-occurrence, which will allow the scientific community to investigate in depth the associations between term pairs like disease-gene and disease-drug. Many drugs were associated with COVID-19, representing the cumulative effort of the scientific community to repurpose existing drugs rather than pursue novel drug discoveries, which is a rational approach in a pandemic situation [74]. We leveraged an automated approach to highlight the effectiveness of drugs against the disease based on sentiment analysis of the text in the literature. Through this literature mining, we found dexamethasone, ivermectin, remdesivir, and others in the list of potential drugs for COVID-19 treatment. We highlighted hydroxychloroquine as an ineffective drug against COVID-19. We extracted disease-gene associations from the literature and, based on cosine similarity against the gold-standard DisGeNET data set, provided a confidence level for the associations between diseases and genes. We found 194 drug-PDB associations, which highlighted the large amount of work performed by the scientific community to understand the mechanism behind drug-target interactions and virus-host protein interaction mechanisms for coronavirus-related diseases. Surprisingly, we found few miRNAs related to COVID-19, indicating the primary focus of the scientific community is toward protein-based drugs rather than RNA-based drugs, though there have been successful RNA-based antiviral drugs. One such drug is Miravirsen, which binds miR-122 to prevent it from hybridizing with the RNA genome of HCV, depriving HCV of its essential cellular cofactor and blocking HCV replication [75]. We expect more research along these lines in the coming months.

### Research Implications

Currently, we are facing the largest public health emergency since the 1918 influenza outbreak [76]. From the beginning of this outbreak, the scientific community has invested large amounts of effort to create vaccines and identify therapeutic solutions. Vaccines for SARS-CoV-2 might come too late to have any effect on the first wave of the COVID-19 pandemic [77]. However, vaccines might be useful in subsequent waves of COVID-19 or in a postpandemic scenario in which COVID-19 becomes a seasonal virus [77]. In this scenario, the identification of drugs with good efficacy and minimal side effects is a rational goal that can be achieved in the near future to combat SARS-CoV-2 [48]. Although promising pharmacological results with repurposed drugs are emerging every day, unfortunately, no drug has been approved thus far for the treatment of COVID-19. Repurposed drugs are under investigation worldwide, many in preclinical and clinical stages [78]. With increasing information about SARS-CoV-2, along with publications about similar respiratory diseases (eg, pneumonia, SARS), it will be essential to investigate existing drugs that are already known to be effective against other respiratory diseases. As a prime example, dexamethasone, an FDA-approved drug, was known to be effective against pneumonia [79], respiratory failure [80], and other diseases. However, there was no evidence of its effectiveness against COVID-19 until its recent breakthrough in a clinical trial [55]. Although final approval of the drug is still pending, had it been investigated earlier, more lives could have been saved.

The research in this study is expected to support the scientific community and decision makers in identifying candidate drugs with proper evidence from the scientific literature. This will also help stakeholders explore existing drugs that are already known to be effective against other respiratory diseases. Although careful manual curation of the identified associations of biomedical entities is the ultimate goal, our novel approach estimates the effectiveness of drugs for coronavirus-related diseases based on natural language processing, sentiment analysis, and deep learning to help the scientific community shorten the potential list of drugs, ultimately saving time and resources.

### Tool and Availability

We made our computational workflow and the resulting database an open source tool named COVID-19Base for use by the scientific community [81,82]. It not only identifies the terms and associations, but also highlights the relevant literature through its digital object identifier (DOI) so that any researcher using this tool can easily check the original source for more detailed information. As the number of scientific publications related to COVID-19 is constantly increasing, we will update the knowledge base on a monthly basis and integrate all recent updates in the knowledge base. COVID-19Base has already gone through its first transformation (from COVID-19Base 1.0 to COVID-19Base 2.0), as the CORD-19 data set was updated during the manuscript preparation phase. The earlier version of the CORD-19 data set contained about 44,000 papers, whereas the current version includes more than 138,000. The knowledge base materials and the source code of our computational approach are available on GitHub [83].

### Limitations

Understandably, our findings as presented in the knowledge base may have some errors due to the inherent limitations of the methods and approaches adopted. This is why the identified inferences and associations are made available to users for review and a feedback mechanism is included in COVID-19Base.

### Conclusions

We proposed a dictionary-based automated computational workflow to find the associations of six different thematic areas related to COVID-19/SARS-CoV-2 and other coronavirus-related diseases in humans. We prepared a knowledge base and made it available as a tool for the scientific community. We believe this knowledge base will help the research community explore the existing drugs and biomedical entities for coronavirus-related diseases, and the lessons learned before this outbreak will allow us to find an effective treatment for COVID-19.

## Abbreviations

ACE2: angiotensin-converting enzyme 2
CORD-19: COVID-19 Open Research Dataset
CRP: C-reactive protein
DNN: deep neural network
DO: Disease Ontology
FDA: Food and Drug Administration
HCV: hepatitis C virus
HGNC: HUGO Gene Nomenclature Committee
miRNA: micro ribonucleic acid
ncRNA: noncoding ribonucleic acid
NIAID: National Institute of Allergy and Infectious Diseases
PDB: Protein Data Bank
POS: part-of-speech
ReLU: rectified linear unit
tf–idf: term frequency–inverse document frequency
